# Looking twice at the gender equity index for public health impact

**DOI:** 10.1186/1471-2458-13-659

**Published:** 2013-07-16

**Authors:** José Fernández-Sáez, Maria Teresa Ruiz-Cantero, Marta Guijarro-Garví, Mercedes Carrasco-Portiño, Victoria Roca-Pérez, Elisa Chilet-Rosell, Carlos Álvarez-Dardet

**Affiliations:** 1 Public Health Research Group, University of Alicante, Alicante, Spain; 2 CIBER Epidemiología y Salud Pública (CIBERESP), Catalonia, Spain; 3 Department of Economics, University of Cantabria, Cantabria, Spain; 4 Department of Obstetrics and Puericulture, Faculty of Medicine, University of Concepcion, Concepcion, Chile; 5 Department of Philosophy of Law and Private International Law, University of Alicante, Alicante, Spain; 6 Grupo de Investigación de Salud Pública. Universidad de Alicante, Edificio Ciencias Sociales, Crta. San Vicente-Alicante s/n. Campus San Vicente del Raspeig. Apartado, Alicante, Postal 99. 03080, Spain

**Keywords:** Gender equity, Index, Education, Empowerment, Income

## Abstract

**Background:**

It has been shown that gender equity has a positive impact on the everyday activities of people (decision making, income allocation, application and observance of norms/rules) which affect their health. Gender equity is also a crucial determinant of health inequalities at national level; thus, monitoring is important for surveillance of women’s and men’s health as well as for future health policy initiatives. The Gender Equity Index (GEI) was designed to show inequity solely towards women. Given that the value under scrutiny is equity, in this paper a modified version of the GEI is proposed, the MGEI, which highlights the inequities affecting both sexes.

**Methods:**

Rather than calculating gender gaps by means of a quotient of proportions, gaps in the MGEI are expressed in absolute terms (differences in proportions). The Spearman’s rank coefficient, calculated from country rankings obtained according to both indexes, was used to evaluate the level of concordance between both classifications. To compare the degree of sensitivity and obtain the inequity by the two methods, the variation coefficient of the GEI and MGEI values was calculated.

**Results:**

Country rankings according to GEI and MGEI values showed a high correlation (rank coef. = 0.95). The MGEI presented greater dispersion (43.8%) than the GEI (19.27%). Inequity towards men was identified in the education gap (rank coef. = 0.36) when using the MGEI. According to this method, many countries shared the same absolute value for education but with opposite signs, for example Azerbaijan (−0.022) and Belgium (0.022), reflecting inequity towards women and men, respectively. This also occurred in the empowerment gap with the technical and professional job component (Brunei:-0.120 vs. Australia, Canada Iceland and the U.S.A.: 0.120).

**Conclusion:**

The MGEI identifies and highlights the different areas of inequities between gender groups. It thus overcomes the shortcomings of the GEI related to the aim for which this latter was created, namely measuring gender equity, and is therefore of great use to policy makers who wish to understand and monitor the results of specific equity policies and to determine the length of time for which these policies should be maintained in order to correct long-standing structural discrimination against women.

## Background

In terms of public health, gender is considered one of the main social determinants of health, together with social class and ethnic group [1]. A gender equity measurement tool is important for health policies and public health surveillance at the national level, since gender equity does not constitute a policy area in its own right and thus its implementation falls mainly within the scope of other policy areas such health and social policies. The fact that States base their policies on equity law implies that all Governments support those who have fewer resources. In other words, the principle of fair treatment is applied in order to improve the skills and abilities of all citizens and thus attain a common level of duties so that all benefit from enhanced well-being. The steps that are being taken towards this form of equality are directly linked to the achievement of the health-focused Millennium Development Goals [2-10]. Another, indirect link with health also exists via the achievement of the education-focused goal [11-14].

The Gender Equity Index (GEI) was launched by Social Watch in 2007 [15] and is aimed at helping to promote gender equity and the autonomy of women [16], which is the third Millennium Development Goal. The GEI has been used both in research [17] and in grey literature [18,19], and has attracted much attention from the media [20]. Institutions such as the World Bank or the Global Development Network have used this new index extensively. The GEI ranks the situation of 157 nations with regard to gender equity in education, economic activity (employment) and empowerment (political participation, representation in government positions, law-making).

Other Indexes include the Gender Development Index (GDI), the Gender Gap Index (GGI) and the Gender Inequality Index (GII). The Gender Development Index, created in 1995 by the UN, is a modified version of the Human Development Index which considers women and men separately for life expectancy at birth and for two important determinants of health, education and income [21]. The Gender Gap Index was introduced by the World Economic Forum in 2006 to measure and monitor the magnitude and scope of gender disparities. This index identifies gender gaps in economic development, education, health, survival and political participation [22].

The Gender Inequality Index, which has been calculated since 2010, shows the loss in human development due to inequality between female and male achievements as regards the dimensions of reproductive health, empowerment and the labour market. Although the GII incorporates empowerment, it also includes a dimension of reproductive health that hinders its association with health variables among women [23], particularly for those of a fertile age. In addition, while the Gender Development Index and the Gender Gap Index consider life expectancy from birth [21,22], this has been replaced in the Gender Equity Index by political participation, making it possible to conduct a better statistical analysis of this index relationship to total and cause-specific mortality, as well as to morbidity.

The GEI was designed to identify inequity solely towards women. The way the GEI is formulated has one drawback which impedes its contribution to raising awareness about human rights in that it only reveals inequity towards women and does not consider those situations where women are relatively better off than men, i.e. inequity towards men [24]. As a result, the index is in conflict with the aims for which it was created. GEI values range from 0 (inequity) to 1 (equity). However, in those situations where the percentage of women (numerator) is greater than the percentage of men (denominator), and the value of the ratio is thus greater than unity, Social Watch equals the gap to 1 [25].

In fact, the greater the denominator with respect to the numerator, the greater the inequity towards women. Furthermore, if the numerator and the denominator coincide, i.e. if the gap is equal to unity, the proportions are maintained and consequently, there is no gender gap, i.e. a situation of equity is reached. However, in those situations where the numerator is greater than the denominator, and therefore the value of the gap is greater than unity (which is possible from an algebraic point of view), Social Watch truncates the result obtained and the value of the gap then equals 1 [25].

The reason behind this lack of attention is the fact that in the majority of societies it is women who traditionally lose out to men not only as regards rights, professional opportunities and responsibilities, but also in relation to participation in resource management and political decision-making processes. In terms of autonomy and capabilities, it is not merely a case of gender still being a conditioning factor in social design, but rather of it being particularly negative for women (less opportunities in education, professional development, lower participation in the labour market and in politics) [26,27]. According to M.C. Nussbaum, in statistical terms women are mainly instruments used by others to achieve their own means rather than agents, i.e. subjects capable of fulfilling their own goals in their own name and in their own right [24,28].

Just as Social Watch developed the GEI to render gender inequities in different countries more visible, in this paper we propose a refined version of the Gender Equity Index that highlights the inequities affecting both women and men, thus generating a more comprehensive measurement of inequity useful in monitoring gender equity for public health surveillance purposes.

## Methods

The methodological proposal for calculating the gender gaps between women and men for the GEI dimensions, termed the modified Gender Equity Index (MGEI) (Table 1), considers situations of gender inequity that are unfavourable towards men and women, applying a methodological change to the definition of the gender gap for the three GEI dimensions. It is aimed at comparing the proportions of women and men with a particular characteristic (c) in absolute terms (difference *P*_*Wc*_ − *P*_*Mc*_), standardising the result so that the “modified gap” (*MGap*. *c* is defined as follows:

$$\mathrm{MGap}.c=\frac{P_{\mathrm{Wc}}-P_{\mathrm{Mc}}}{P_{\mathrm{Wc}}+P_{\mathrm{Mc}}}$$

**Table 1:** Method used to calculate gender gaps in the Gender Equity Index (proposed by Social Watch) and the modified Gender Equity Index

**Social watch gender equity index (SW)**	**Modified gender equity index**
SW defines the corresponding gap between women (W) and men (M) as: $\mathrm{Gap}.c=\frac{\%\mathrm{Wc}}{\%\mathrm{Mc}}.\mathrm{WF}P^{-1}$ where % *Wc* and % *Mc* are, respectively, the% of W and M with the characteristic	The MGEI compares the proportions of women and men with a particular characteristic (c) in absolute terms (difference *P*_*Wc*_ − *P*_*Mc*_), standardising the result so that the “modified gap” (*MGap*. *c*) is defined as:
(c), and $\mathrm{WFP}=\frac{\mathrm{Po}p_{W}}{\mathrm{Po}p_{M}}$ is the weight of the female population.	$$\mathrm{MGap}.c=\frac{P_{\mathrm{Wc}}-P_{\mathrm{Mc}}}{P_{\mathrm{Wc}}+P_{\mathrm{Mc}}}$$
This expression of the gender gap is simplified by replacing the inverse value of the weight of the female population: $\begin{array}{l} \mathrm{Gap}.c=\frac{\%\mathrm{Wc}}{\%\mathrm{Mc}}.\frac{\mathrm{Po}p_{M}}{\mathrm{Po}p_{W}}=\frac{\mathrm{Wc}.100/\mathrm{Pop}}{\mathrm{Mc}.100/\mathrm{Pop}}.\frac{\mathrm{Po}p_{M}}{\mathrm{Po}p_{W}}\\ =\frac{\mathrm{Wc}}{\mathrm{Mc}}.\frac{\mathrm{Po}p_{M}}{\mathrm{Po}p_{W}}=\frac{\mathrm{Wc}}{\mathrm{Po}p_{W}}.\frac{\mathrm{Po}p_{M}}{\mathrm{Wc}} \end{array}$ Where *Pop* is the total population.	The proportions have values of between 0 and 1, from which it results that: − (*P*_*Wc*_ + *P*_*Mc*_) ≤ *P*_*Wc*_ − *P*_*Mc*_ ≤ *P*_*Wc*_ + *P*_*Mc*_, whilst dividing by *P*_*Wc*_ + *P*_*Mc*_ results in − 1 ≤ *MGap*. *c* ≤ 1.
If $P_{\mathrm{Mc}}=\frac{\mathrm{Wc}}{\mathrm{Po}p_{W}}$ and $P_{\mathrm{Mc}}=\frac{\mathrm{Mc}}{\mathrm{Po}p_{M}}$ are respectively the proportions of W and M with a particular characteristic (c) out of their corresponding totals, the gap suggested by SW allows the following formulation: $$\mathrm{Gap}.c=\frac{P_{\mathrm{Wc}}}{P_{\mathrm{Mc}}}$$	
Once the gender gap has been calculated, the GEI is calculated as the arithmetic mean of the 3 gaps: $\mathrm{GEI}=\frac{\mathrm{Empowerment}.\mathrm{Gap}+\mathrm{Economic}.\mathrm{Act}.\mathrm{Gap}.+\mathrm{Education}.\mathrm{Gap}}{3}$	
Social Watch GEI values: 0 (inequity) - 1 (gender equity)	Modified GEI values: - 1 (Women inequity) 0 (equity) 1 (Men inequity)

The proportions have values of between 0 and 1, from which it results that: − (*P*_*Wc*_ + *P*_*Mc*_) ≤ *P*_*Wc*_ − *P*_*Mc*_ ≤ *P*_*Wc*_ + *P*_*Mc*_, whilst dividing by *P*_*Wc*_ + *P*_*Mc*_ results in − 1 ≤ *MGap*. *c* ≤ 1.

The GEI values are positive, and vary between 0 (gender inequity towards women) and 1 (gender equity), whereas the interpretation of the modified gap is the following:

●  If *MGap*. *c* = 0, the numerator of the gap equals 0, with both proportions coinciding, and a situation of EQUITY is reached, since there is no disparity between women and men for the characteristic c.

●  If *MGap*. *c* = − 1, then *P*_*Wc*_ − *P*_*Mc*_ = − (*P*_*Wc*_ + *P*_*Mc*_) and therefore *P*_*Wc*_ = 0, this indicates a situation of MAXIMUM INEQUITY towards women. Negative gap values reflect the existence of inequity towards women, and the closer the gap is to −1, the greater this inequity becomes.

●  If *MGap*. *c* = 1, then *P*_*Wc*_ − *P*_*Mc*_ = *P*_*Wc*_ + *P*_*Mc*_ and consequently, *P*_*Mc*_ = 0, indicating a situation of MAXIMUM INEQUITY towards men. Positive gap values reveal the existence of inequity towards men, which increases the closer the gap value is to 1.

Moreover, interpreting the gap in absolute terms enables the distance between both genders to be measured: gap values close or equal to 0 indicate an absence of distance (equity), whereas the closer the values become to unity, the greater the gap between both genders for the characteristic considered (inequity).

### Measuring gender equity in 2005 using the MGEI

Using information sources provided by Social Watch [24], the MGEI for 2005 was calculated for the 114 countries with the available data necessary for obtaining the modified gaps in education, economic activity and empowerment (Appendix 1). In order to highlight the loss of information caused by truncating the GEI value and the corresponding gap value of the three dimensions to 1, and to illustrate the interpretative differences stemming from the two different methodologies, those countries for which the MGEI has the same absolute value but with a different sign (plus or minus), and consequently opposing inequity, were identified.

As the GEI and the MGEI are ordinal measurements that allow countries to be ranked, calculating the Spearman’s rank coefficient from country rankings obtained according to both indexes constituted the best means to evaluate the level of concordance between both classifications, and consequently, the degree of similarity between the two gender equity measurement methods. Similarly, the values of this coefficient were also calculated from the country rankings obtained from the gap and the modified gap for education, economic activity and empowerment.

To compare the degree of sensitivity and obtain the inequity given by the two methods, the variation coefficient of the GEI and MGEI values was calculated, as well as the variation coefficient of the gap and modified gap values for education, economic activity and empowerment. A higher variation coefficient indicates greater dispersion of the index values (and their gaps) and therefore represents a more sensitive index for measuring inequity.

## Results

**Figure 1:**
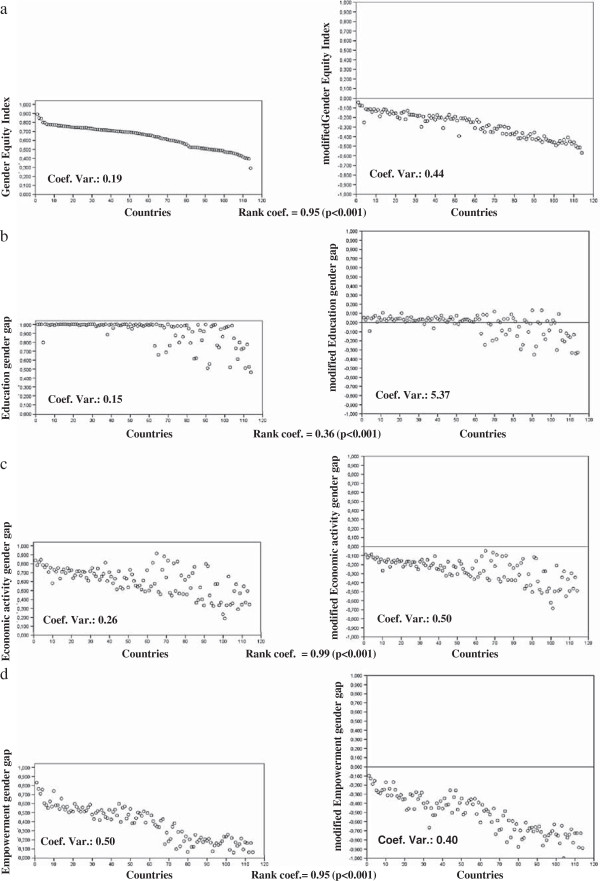
Distribution of Gender equity index (GEI) and modified Gender equity index (MGEI) values in 114 countries and their corresponding components (Education gender gap, Economic activity gender gap and Empowerment gender gap).

The graphs in Figure 1 show the distribution of the GEI values and their respective component gaps (graphs in the left column) and the MGEI values and their corresponding component gaps (graphs in the right column) for 114 countries. The profile of the two graphs in 1a shows that both methods yielded a similar country ranking (Spearman’s rank coefficient estimator equal to 0.95, p < 0.001). However, the GEI values (variation coefficient: 0.19) presented less variability than those of the MGEI (variation coefficient: 0.44).

As regards the education gender gap, the rank coefficient between both methods was low (0.36). Thus, the methods ranked the countries differently for the education gender gap. In Figure 1b, it can be observed that most countries obtained a gap value equal to 1 (equity) due to the GEI truncation method, whereas the MGEI method showed that inequity towards men existed in 65.8% of the countries analysed (modified education gap greater than 0) (Figure 1b). Consequently, the education gender gap values showed less dispersion (variation coefficient of 0.15) than those of the modified education gender gap (variation coefficient of 5.37).

Since no gap values were truncated at 1, the GEI and MGEI methodologies ranked the studied countries in a very similar manner as regards gender gaps in economic activity and empowerment (rank coefficient: 0.99 and 0.95, respectively, p < 0.001). (Figure 1c and d).

According to the GEI method, the 25 countries listed in Table 2 were considered to be equitable in terms of education, since the values of the corresponding gender gaps were truncated to 1 (1st column). However, the inequity towards men that is masked by this procedure was revealed in the values higher than 0 for the modified gender gap obtained for these countries (3rd column). Furthermore, some countries may register the same degree of inequity in education, that is, the same absolute value of the modified education gap, but with different signs (minus or plus), which means that the inequity in education is suffered by women or men, respectively. This was the case of Chile (−0.009) and Greece (0.009), Swaziland and Peru (−0.011) and Ukraine (0.0011), Hong Kong and China (−0.019) and El Salvador (0.019), Azerbaijan (−0.022) and Belgium (0.022), and Vietnam (−0.063) and Panama (0.063) (see Appendix).

**Table 2:** Comparison of the education gap obtained using the gender equity index and that obtained by the modified gender equity index, in educationally equitable countries according to Social Watch (SW, 2005)

**Country**	SW education gap*	SW education gap without cut-off**	Modified education gap***
Austria	1.000	1.038	0.016
Australia	1.000	1.048	0.020
Belgium	1.000	1.050	0.022
Finland	1.000	1.063	0.029
France	1.000	1.070	0.030
Slovakia	1.000	1.073	0.032
Costa Rica	1.000	1.078	0.035
Canada	1.000	1.085	0.036
Israel	1.000	1.085	0.036
Brazil	1.000	1.088	0.037
Ireland	1.000	1.088	0.039
Poland	1.000	1.098	0.040
Philippines	1.000	1.090	0.041
USA	1.000	1.103	0.043
Denmark	1.000	1.105	0.044
UK	1.000	1.105	0.044
Norway	1.000	1.138	0.054
Cuba	1.000	1.168	0.060
Latvia	1.000	1.190	0.067
Honduras	1.000	1.178	0.075
Dominican Republic	1.000	1.200	0.078
Lesotho	1.000	1.190	0.084
Uruguay	1.000	1.295	0.102
Barbados	1.000	1.368	0.106
Jamaica	1.000	1.370	0.120

*Range: 0 to 1 (1 = equity), **range: 0 to ∞, ***range: -1 to 1 (0 = equity).

Table 3 shows the gender gap values for the different education components. For literacy, the gap values obtained using the GEI and MGEI methods led to an identical interpretation, except for 5 countries (Uruguay, Honduras, Philippines, Jamaica and Lesotho), since the GEI method truncated the values at 1 (indicating equity), whilst the modified index showed inequity towards men (Table 3a). In primary education (Table 3b), both methods provided identical results (equity in 9 countries and inequity suffered by women in 15 countries), except for Israel, for which the gap was truncated at 1, whilst the modified index showed inequity towards men. Table 3c shows the values of the secondary education gender gap. Although both indexes detected equity in Barbados, France and Cuba and inequity suffered by women in 6 countries, only the modified index registered inequity towards men in 16 countries. In university education, the gaps registered for all the countries would have exceeded unity using the GEI method if they had not been truncated. Using the MGEI method, all the countries obtained a positive gap, since women outnumbered men in terms of university enrolment (Table 3d).

**Table 3:** **Comparison of the education gap components**^**a**^**obtained using the gender equity index and those obtained by the modified gender equity index, in educationally equitable countries according to Social Watch (SW, 2005)**

**2.a Countries**	SW literacy gap*	Modified literacy gap**	**2.b Countries**	SW 1º gap*	Modified 1º gap**
Finland	1.000	0.000	Brazil	0.930	−0.036
Norway	1.000	0.000	Cuba	0.950	−0.026
Denmark	1.000	0.000	Dominican R	0.950	−0.026
Barbados	1.000	0.000	Latvia	0.960	−0.020
Australia	1.000	0.000	Uruguay	0.980	−0.010
Latvia	1.000	0.000	Finland	0.990	−0.005
Canada	1.000	0.000	Australia	0.990	−0.005
USA	1.000	0.000	Philippines	0.990	−0.005
UK	1.000	0.000	USA	0.990	−0.005
Slovakia	1.000	0.000	Slovakia	0.990	−0.005
Belgium	1.000	0.000	Belgium	0.990	−0.005
Austria	1.000	0.000	France	0.990	−0.005
France	1.000	0.000	Poland	0.990	−0.005
Israel	1.000	0.000	Ireland	0.990	−0.005
Poland	1.000	0.000	Costa Rica	0.990	−0.005
Ireland	1.000	0.000	Norway	1.000	0.000
Cuba	1.000	0.000	Denmark	1.000	0.000
Brazil	1.000	0.000	Barbados	1.000	0.000
Costa Rica	1.000	0.000	Canada	1.000	0.000
Dominican R	1.000	0.000	UK	1.000	0.000
Uruguay	1.000	0.005	Austria	1.000	0.000
Honduras	1.000	0.005	Honduras	1.000	0.000
Philippines	1.000	0.010	Lesotho	1.000	0.000
Jamaica	1.000	0.074	Jamaica	1.000	0.000
Lesotho	1.000	0.103	Israel	1.000	0.005
**2.c Countries**	2º gap*	modified 2º gap**	**2.d Countries**	3º gap*	modified 3º gap**
Australia	0.950	−0.026	Austria	1.000	0.091
Austria	0.950	−0.026	Finland	1.000	0.095
Belgium	0.970	−0.015	Philippines	1.000	0.103
Canada	0.980	−0.010	Belgium	1.000	0.107
Israel	0.990	−0.005	Australia	1.000	0.111
Poland	0.990	−0.005	Costa Rica	1.000	0.115
Barbados	1.000	0.000	Ireland	1.000	0.119
France	1.000	0.000	Lesotho	1.000	0.119
Cuba	1.000	0.000	Slovakia	1.000	0.127
Norway	1.000	0.005	France	1.000	0.127
Latvia	1.000	0.005	Brazil	1.000	0.138
Slovakia	1.000	0.005	Israel	1.000	0.145
USA	1.000	0.010	Canada	1.000	0.153
Denmark	1.000	0.015	Denmark	1.000	0.163
UK	1.000	0.015	UK	1.000	0.163
Jamaica	1.000	0.015	USA	1.000	0.167
Finland	1.000	0.024	Poland	1.000	0.170
Costa Rica	1.000	0.029	Honduras	1.000	0.187
Ireland	1.000	0.043	Norway	1.000	0.213
Brazil	1.000	0.048	Dominican R	1.000	0.242
Philippines	1.000	0.057	Cuba	1.000	0.265
Uruguay	1.000	0.074	Latvia	1.000	0.283
Dominican R	1.000	0.095	Uruguay	1.000	0.340
Honduras	1.000	0.107	Jamaica	1.000	0.392
Lesotho	1.000	0.115	Barbados	1.000	0.424

^a^Literacy Values, in primary (1º), secondary (2º) and university (3º) education. *range: 0 to 1 (1 = equity), **range: -1 to 1 (0 = equity).

In Table 4 it can be observed that some countries shared the same degree of inequity, represented by the same absolute value of the modified gap for all education components, but towards opposite sexes. For example, in university studies, Guatemala presented an inequity gap (−0.163) towards women, whilst Denmark and the United Kingdom showed the same degree of inequity (0.163) but towards men.

**Table 4:** **Countries with the same absolute value of inequity in the education gap components**^**a **^**obtained using the modified gender equity index, highlighting cases of inequity towards women (− values) or men (+ values)**

**Countries**	**Values**	**Countries**	Values*
	**Literacy**		
Albania, Azerbaijan, Bulgaria, United Arab Emirates, Kyrgyzstan, Moldova, Panama, Qatar, Trinidad Tobago, Ukraine, Venezuela, Italy, Samoa	**−0.005**	Honduras, Uruguay	**0.005**
Croatia, Paraguay, Romania	**−0.010**	Botswana, Philippines	**0.010**
Mexico, Swaziland	**−0.015**	Malta	**0.015**
	**Primary**		
Albania, Argentina, Australia, Belgium, Bahrain, Bulgaria, China, Costa Rica, Croatia, Slovakia, Slovenia, Philippines, Finland, France, Ireland, Italy, Kazakhstan, Kyrgyzstan, Moldova, Poland, Qatar, Romania, USA	**−0,005**	Israel, Jordan, Mauritania, Namibia	**0.005**
Spain, Azerbaijan, Botswana, Colombia, Mexico, The Netherlands, Uruguay, Venezuela, Zimbabwe	**−0.010**	Mongolia, Rwanda	**0.010**
Belarus, United Arab Emirates, Estonia, Iceland, Nicaragua, Panama, Paraguay, Trinidad and Tobago, Tunisia	**−0.015**	Bangladesh	**0.015**
Russian Federation, Indonesia, Israel, Italy, Mauritius , Poland, Latvia	**−0.005**	Belarus, Chile, Slovakia, Estonia, Kyrgyzstan, Latvia, Norway, Peru, Romania	**0.005**
Canada, Greece, Macedonia, Netherlands, Qatar	**−0.010**	Cyprus, Croatia, Jordan, Paraguay, USA	**0.010**
Belgium, Kazakhstan, Vietnam	**−0.015**	Bangladesh, Denmark, El Salvador, Iceland, Jamaica, Malta, Moldova, UK	**0.015**
Albania, Saudi Arabia, Azerbaijan, Hong Kong, Oman, Swaziland	**−0.020**	Brunei Darussalam, Trinidad and Tobago	**0.020**
	**University**		
Chile	**−0.020**	Cape Verde	**0.020**
Guatemala	**−0.163**	Denmark, UK	**0.136**
Vietnam	**−0.170**	Argentina, Poland	**0.170**

^a^Literacy Values , in primary, secondary and university education.

*Countries with positive values according to the modified gender gap index have the value of 1 in the education gap components identified according to the method of Social Watch.

Similarly, 62 countries presented the same phenomenon for the empowerment gender gap component, Technical and Professional Jobs. Thus, Austria, Belgium and Greece obtained −0.020, indicating an inequity gap toward women, whilst Botswana, Panama, the Dominican Republic, Sweden and Vietnam obtained 0.020, indicating an inequity gap toward men; likewise, Spain obtained −0.040 whilst Barbados, Honduras, Brazil, Ireland, Macedonia, Chile and China obtained 0.040; France and the United Kingdom obtained −0.060 whilst Argentina, Denmark, New Zealand and Trinidad Tobago obtained 0.060; Italy and Peru obtained −0.080 whilst Israel, Mongolia, Paraguay, Thailand and Uruguay obtained 0.080; Cyprus and El Salvador obtained −0.100 whilst Finland and Namibia obtained 0.100; Brunei obtained −0.120 whilst Australia, Canada, Iceland and the U.S.A. obtained 0.120; Mauritius obtained −0.140 whilst Slovenia, Kyrgyzstan and Romania obtained 0.140); Mexico obtained −0.160 whilst Slovakia obtained 0.160; Costa Rica, Hong Kong and Malaysia obtained −0.200 whilst Bulgaria obtained 0.200; Malta obtained −0.240 whilst Cuba and Hungary obtained 0.240; Morocco obtained −0.300 whilst the Russian Federation and Latvia obtained 0.300; Iran obtained −0.320 whilst Moldova obtained 0.320; Cambodia and Oman obtained −0.340 whilst Kazakhstan and Lithuania obtained 0.340; and Ethiopia obtained −0.400 whilst Estonia obtained 0.400. A similar result was obtained for Latvia and the U.S. (−0.160) and the Philippines (0.160) in relation to another of the empowerment components: law-making.

## Discussion

Based on considerations related to social justice, upon which public health rests, and the measurement and data analysis instruments used for the design and application of said policies, we strongly suggest that the GEI should be reformulated. Although the country rankings obtained using the MGEI and the GEI were quite similar, the MGEI corrected the bias produced in inequity measurements obtained using the GEI, which truncates all values above unity to 1. However, the reformulation of the GEI proposed here does not negate the need to eradicate those cases which are evidently unjust towards women. Rather, it is aimed at achieving the goal of eradicating this injustice by using mechanisms which are based on the principle of equal consideration for all people regardless of their gender, thus measuring all inequalities independently of the gender affected (female or male). The gender sensitivity of the MGEI could render this index a useful source of data for the purposes of developing future public policies in pursuance of equality: this tool will enable surveillance of areas of inequality by gender, might prove the coexistence of different areas in which men and women respectively experience inequality in the same country and will allow further research on the possible causal connections. It is important for indicators to be sensitive to changes in gender equity values and to detect shortcomings in the education, economic activity (employment) and empowerment of women or men so that immediate action can be taken, since these are the basic and interdependent cornerstones of social development, and if one of them is weakened, the entire structure may collapse.

The limitations of this study include the MGEI calculations for certain countries, due to a lack of available data from the information sources employed by Social Watch [24,25,29]. Consequently, it was not possible to carry out a comparative analysis of all of the 157 countries for which the GEI is calculated. However, the values of the MGEI and of the corresponding education and income gaps showed greater dispersion than those of the GEI, indicating that the proposed method is a more accurate tool for identifying situations of inequity [30-32].

The MGEI can easily be interpreted since the 0 value indicates equity due to a lack of distance between the sexes in the overall values obtained from the combination of its dimensions. Moreover, it indicates in which sense inequity is produced by varying its range of values between −1 (inequity towards women) and 1 (inequity towards men).

Our findings show that education is the GEI dimension most affected by truncating all values greater than unity to 1. This occurred for 25 developed and developing countries and masks inequity towards men as regards education. Thus, although the data on gender equity in economic activity and empowerment were very similar using either the GEI or the MGEI method, this was not the case for equity in education. In comparison with the GEI, the MGEI revealed two new findings. On the one hand, inequity towards men in education was present in more countries, and on the other hand, when inequity was present towards women, the gender gap was much greater. These are extremely important findings, since more efficient management of State funds for education, aimed at avoiding early school leaving for example, could help forestall the difficulties such students would otherwise encounter in finding employment and thus prevent their eventual marginalisation [33]. Furthermore, some countries presented the same absolute value and therefore the same level of inequity in education, but in an opposite direction, showing inequity towards women with a negative value or inequity towards men with a positive value. With the GEI, this information is lost by truncating at 1. It should be noted that not every country with inequity is a country with unfair policies. It is necessary to confirm whether this outcome is due to a set of temporary circumstances or to a shift in a particular status quo that had previously been unfavourable towards women. If results from several years were to indicate that a particular sector or sphere had established structural inequity towards men, this could be corrected by means of State intervention. Moreover, in university education, a greater number of female students does not necessarily represent a higher number of qualified women working in positions in accordance with their qualification. Therefore, in order to achieve systemic equity among professional adults, unequal access to university education must be allowed in favour of women over a certain period of time, whether this be spontaneous or by means of quotas, incentives or grant policies.

The notion of empowerment in the GEI formula involves bringing the value of autonomy to the foreground and embracing the ultimate goal of gender equality policies. Thus, it recognizes the value of an empowered individual not so much a subject entitled to well-being, but rather as an agent with his/her own skills, values, judgments and priorities. Empowerment also involves accepting the need for public policies aimed at building political communities where all citizens participate in designing the social framework and fabric [29]. With the MGEI method, some countries obtained the same degree of inequity for empowerment components (Technical and Professional Jobs, and Law-making) as the GEI. However, whilst in many countries this inequity was shown towards women, in quite a few others the inequity was towards men. Therefore, for many years to come and in most societies, public policies aimed at empowerment will still necessarily focus on women, but the final target group will include everyone, regardless of gender [29]. It must be highlighted that gender equity continues despite the economic development of a particular society. Social Watch [24] and studies by Nussbaum and Sen [34,35] have demonstrated the lack of a direct relationship between wealth and equity. However, the MGEI revealed that some countries identified by the World Bank as having a high economic level, such as India, presented high levels of gender inequity, whereas others with low levels of wealth, such as Lithuania, have taken significant steps towards achieving gender equity.

## Conclusion

Given that the value under scrutiny is equity, an attempt has been made to refine an index that could be particularly useful for the surveillance of women’s and men’s health as well as for future health policy initiatives. The index produces gender-sensitive values that that will make possible (1) to monitor the results of specific policies, (2) to assess the influence of these policies on health and illness indicators for both sexes, and (3) to determine the length of time for which these policies should be maintained in order to correct long-standing structural discrimination against women. The index also enables efforts to correct inequities towards men. Moreover, the negative impact that a particular Public Health Policy may have on men or women will be revealed by the MGEI values, enabling such policies to be redefined or abandoned when the time comes.

## Competing interests

The authors declare that they have no competing interests.

## Authors’ contributions

JFS conceived the study and participated in study design, data acquisition, calculation of indexes, data analysis and coordination and drafting of the paper. MTR participated in study design and data acquisition, and helped to draft the manuscript. MGG participated in the calculation of indexes and helped to draft the paper. MCP participated in study design and discussion of results. VRP, CAD and ECR made intellectual and theoretical contributions to the interpretation of the results. All authors read and approved the final manuscript.

## Pre-publication history

The pre-publication history for this paper can be accessed here:

http://www.biomedcentral.com/1471-2458/13/659/prepub
